# Immune Checkpoint Inhibitor-Induced Myasthenia Gravis

**DOI:** 10.3389/fneur.2020.00634

**Published:** 2020-07-16

**Authors:** Yi-Te Huang, Ya-Ping Chen, Wen-Chih Lin, Wu-Chou Su, Yuan-Ting Sun

**Affiliations:** ^1^Department of Neurology, National Cheng Kung University Hospital, College of Medicine, National Cheng Kung University, Tainan, Taiwan; ^2^Division of Hematology and Oncology, Department of Internal Medicine, National Cheng Kung University Hospital, College of Medicine, National Cheng Kung University, Tainan, Taiwan; ^3^Department of Physical Medicine and Rehabilitation, Chi Mei Medical Centre—Chiali Branch, Tainan, Taiwan; ^4^Advanced Optoelectronic Technology Center, National Cheng Kung University, Tainan, Taiwan

**Keywords:** myasthenia gravis, immune-related adverse events, cancer therapy, neuromuscular junction disorder, immune checkpoint inhibitor

## Abstract

The development of immune checkpoint inhibitors (ICIs) has been a major breakthrough in cancer immunotherapy. The increasing use of ICIs has led to the discovery of a broad spectrum of immune-related adverse events (irAEs). Immune-related myasthenia gravis (irMG) is a rare but life-threatening irAE. In this review, the clinical presentations of irMG are described and the risk of irMG-related mortality is examined using information from relevant studies. In 47 reported cases of irMG with clear causes of mortality, irMG appeared to be a distinct category of neuromuscular disorders and differed from classical MG in terms of its demographic patient characteristics, pathogenesis, serology profile, response to treatment, associated complications, and prognosis. Because of the high mortality of irMG, measures to increase the vigilance of medical teams are necessary to ensure the timely identification of the signs of irMG and early treatment, particularly in the early course of ICI therapy. The diagnostic plans should be comprehensive and include the evaluation of other organ systems, such as the dermatological, gastrointestinal, respiratory, neuromuscular, and cardiovascular systems, in addition to the traditional diagnostic tests for MG. Treatment plans should be individualized on the basis of the extent of organ involvement and clinical severity. Additional therapeutic studies on irMG in the future are required to minimize irAE-related mortality and increase the safety of patients with cancer in the ICI era.

## Introduction

Immune checkpoint inhibitors (ICIs) are agents that release the brakes on the immune system, which identifies and eliminates cancer cells. ICIs were developed to treat advanced-stage malignancy and have recently gained importance in oncology (1). The increasing use of ICIs has led to the discovery of diverse and sometimes fatal immune-related adverse events (irAEs) worldwide, which involve multiple organ systems, including the dermatological, gastrointestinal, respiratory, neuromuscular, and cardiovascular systems (1). ICI-related neurological complications include encephalitis, seizure, leukoencephalopathy, myelopathy, polyneuropathy, myasthenia gravis (MG), and myositis. Immune-related MG (irMG) is a rare but life-threatening complication, unlike classical MG which is a relatively benign disorder. Because irMG has a broad clinical spectrum of severity and is associated with high mortality, this review focused on studies relevant to irMG and had two main goals: first, to describe the clinical presentations of irMG and compare them with classical MG and, second, to identify factors that are associated with irMG-related mortality. The findings of the present review can facilitate the timely identification and treatment of irMG, which is a life-threatening complication in the ICI era.

## Method

### Literature

We used the following terms, “immune checkpoint inhibitor” AND “neurological” OR “myasthenia gravis” OR “neuromuscular,” in our search strategy to identify published cases of ICI-induced MG in the PubMed database until March 1, 2020. We evaluated articles, including case reports and case series, in English that provided original patient data. Among the 92 articles found on PubMed, 45 articles (49 cases, one case of our own) were included in the review (Supplementary Table 1) (2–45). After reading each article, the cases with several missing data or undetermined causes of mortality were excluded. Finally, 47 cases were selected for analysis. The following parameters of the cases were analyzed: age, sex, cancer type, ICI type, onset time of irMG symptoms after the initiation of ICI therapy, symptoms of irMG, results of diagnostic tests, treatments for irMG, and involvement of other organ systems. Organ systems with corresponding irAEs reported by Society for Immunotherapy of Cancer include skin, gut, endocrine, lung, musculoskeletal, cardiovascular, hematologic, renal, neurologic, and ophthalmologic systems (1). In this review, various components of the neuromuscular system were coded as neuromuscular junction disorder, myositis, and peripheral neuropathy because the risk of mortality may be varied, while neuropathy or myositis appears concomitantly with irMG. The causes of death were determined according to the descriptions in the reports. None of the cases underwent autopsy. The irMG-related death included hypercapnic respiratory failure and aspiration pneumonia due to bulbar paralysis.

### Statistics

The data were analyzed statistically by using SPSS (version 24.0, IBM Corporation, USA). Mann–Whitney *U*-test or chi-square or Fisher's exact test was applied depending on the type of data. Student's *t*-test was applied after the data passed the normality test. The data were presented as mean ± standard deviation (SD). Values of *P* < 0.05 were considered as statistically significant.

## Results

### General Features of irMG

#### Demographic Data, Diagnostic Tests, and Symptoms of irMG

Among the included 47 cases (27 male) of irMG, the age of onset was 72.9 ± 10.0 years; 14.9 and 85.1% of the cases exhibited the ocular and the generalized types of irMG, respectively (Table 1). The overall mortality rate was 44.7% (21/47) and the irMG-related mortality rate was 29.8% (14/47). The irMG-related mortality rate was much higher than that of classical MG, which has been reduced to 6–8% after introducing immunosuppressants as standard treatments.

**Table 1:** Summary of demographic data, clinical features, and treatment choice of immune-related myasthenia gravis.

	**Total (*N* = 47)**	**Mortality relevant to myasthenia gravis (MG) (*N* = 14)**	**Immortality or mortality irrelevant to MG (*N* = 33)**	***P*-value**
Demographic data				
Age, years, range (mean ± SD)	34–87 (72.9 ± 10.0)	63–85 (73.6 ± 6.7)	34–87 (72.5 ± 11.2)	0.88
Sex, % male	27/47 (56.3%)	9/14 (64.3%)	18/33 (54.5%)	0.768
Cancer type (%)				
Melanoma	23/47 (48.9%)	7/14 (50%)	16/33 (48.5%)	1.000
Small-cell lung carcinoma	5/47 (10.6%)	3/14 (21.4%)	2/33 (6.1%)	0.296
Non-small-cell lung carcinoma	7/47 (14.9%)	0/14 (0%)	7/33 (21.2%)	0.156
Squamous cell carcinoma	3/47 (6.4%)	1/14 (7.1%)	2/33 (6.1%)	1.000
Renal cell carcinoma	5/47 (10.6%)	3/14 (21.4%)	2/33 (6.1%)	0.296
Others	4/47 (8.5%)	0/14 (0%)	4/33 (12.1%)	0.429
Immune checkpoint inhibitor (ICI) drug choice				
Cytotoxic-lymphocyte-associated protein 4 inhibitors				
Ipilimumab (%)^a^	7/47 (14.9%)	2/14 (14.3%)	5/33 (15.2%)	1.000
Tremelimumab (%)^b^	1/47 (2.1%)	1/14 (7.1%)	0/33 (0%)	0.655
Programmed death protein 1 inhibitors				
Nivolumab (%)^a^	23/47 (48.9%)	9/14 (64.3%)	14/33 (42.4%)	0.293
Pembrolizumab (%)	19/47 (40.4%)	4/14 (28.6%)	15/33 (45.5%)	0.451
Programmed death-ligand 1 inhibitors				
Durvalumab (%)^b^	1/47 (2.1%)	1/14 (7.1%)	0/33 (0%)	0.655
ICI combination therapy	4/47 (8.5%)	3/14 (21.4%)	1/33 (3.0%)	0.135
MG features				
Previous MG diagnosis (%)	9/47 (19.1%)	3/14 (21.4%)	6/33 (18.2%)	1.000
Onset time, week (mean ± SD)	1–12 (4.9 ± 2.80)	1–10 (4.0 ± 2.79)	2–12 (5.3 ± 2.75)	0.0302^*^
Onset time, cycle (mean ± SD)	1–4 (2.00 ± 0.86)	1–3 (1.71 ± 0.73)	1–4 (2.12 ± 0.89)	0.152
Presenting symptoms				
Ocular	37/47 (78.7%)	11/14 (78.6%)	26/33 (78.8%)	1.000
Bulbar	18/47 (38.3%)	5/14 (35.7%)	13/33 (39.4%)	1.000
Generalized	27/47 (57.4%)	8/14 (57.1%)	19/33 (57.6%)	1.000
Respiratory	19/47 (40.4%)	7/14 (50.0%)	12/33 (36.4%)	0.585
Diagnostic tests (positive/checked)				
Ice packing test	3 (3/3 = 100%)	0 (0/0)	3 (3/3 = 100%)	NA
Edrophonium test	4 (4/6 = 66.7%)	2 (2/2 = 100%)	2 (2/4 = 50.0%)	0.759
AChR Ab	30 (30/45 = 66.7%)	7 (7/13 = 53.8%)	23 (23/32 = 71.9%)	0.416
MuSK	1 (1/19 = 5.3%)	0 (0/6 = 0%)	1 (1/13 = 7.7%)	1.000
Repetitive nerve stimulation test	11 (11/23 = 47.8%)	4 (4/6 = 66.7%)	7 (7/17 = 41.2%)	0.549
Treatment for MG				
Intravenous immunoglobulin	21	7/14 (50%)	14/ 33 (42.4%)	0.875
Plasma exchange	18	7/14 (50%)	11/33 (33.3%)	0.455
Methylprednisolone (high)	14	4/14 (28.6%)	10/33 (30.3%)	1.000
Methylprednisolone (low)	12	6/14 (42.9%)	6/33 (18.2%)	0.159
Prednisone	15	7/14 (50%)	19/33 (57.6%)	0.875
Pyridostigmine	21	7/14 (50.0%)	14/33 (42.4%)	0.875
Rituximab	2	0/14 (0%)	2/33 (6.1%)	0.88
Associated complications				
Skin (erythema, pruritus)	2/47 (4.3%)	0/14 (0%)	2/ 33 (6.1%)	0.88
Digestive organ (hepatitis)	3/47 (6.4%)	2/14 (14.3%)	1/33 (3.0%)	0.429
Endocrine (hypophysitis)	1/47 (2.1%)	1/14 (7.1%)	0/33 (0%)	0.655
Respiratory (pneumonitis)	5/47 (10.6%)	2/14 (14.3%)	3/33 (9.1%)	1.000
Joint (arthritis)	3/47 (6.4%)	1/14 (7.1%)	2/33 (6.1%)	1.000
Heart (myocarditis)	11/47 (23.4%)	6/14 (42.9%)	5/33 (15.2%)	0.357
Blood (leukopenia)	2/47 (4.3%)	0/14 (0%)	2/33 (6.1%)	0.88
Muscle (myositis)	23/47 (48.9%)	8/14 (57.1%)	15/33 (45.5%)	0.679
Nerve (peripheral neuropathy)	4/47 (8.5%)	2/14 (14.3%)	2/33 (6.1%)	0.724
Kidney (renal failure)	1/47 (2.1%)	1/14 (7.1%)	0/33 (0%)	0.655
Eye (uveitis)	0/47 (0%)	0/14 (0%)	0/33 (0%)	NA
Averaged number of organ system involvement	1.15	1.5	1.0	0.038^*^
Others (creatine kinase)	27/47 (57.4%)	9/14 (64.3%)	18/33 (54.5%)	0.768

a *Three patients received combination therapy of ipilimumab and nivolumab*.

b *One patient received a combination therapy of tremelimumab and durvalumab*.

*NA, not available*.

* *p < 0.05, one-tail t-test*.

The commonly used diagnostic tools for classical MG, such as the repetitive nerve stimulation test (RNST), the pyridostigmine/edrophonium test, and autoantibody serological tests, were not used to test all 47 cases of irMG. The serological test for the anti-acetylcholine receptor (AChR) antibody was the most commonly used test (45/47 = 95.7%), followed by the RNST (23/47 = 48.9%), the test for the anti-muscle specific kinase (MuSK) antibody, the pyridostigmine/edrophonium test, and the ice pack test. The positive rates of anti-AChR antibody and anti-MuSK antibody tests among patients with irMG were 66.7% (30/45 = 66.7%) and 5.3% (1/19 = 5.3%), respectively. About half of the patients who underwent the RNST showed positive results (11 of 23 cases had undergone RNST) and 66.7% of patients who underwent pyridostigmine/edrophonium testing showed positive results (four of six cases had undergone the pyridostigmine/edrophonium test; Table 1).

The presentation of symptoms of irMG includes exercise intolerance in the ocular (ptosis and diplopia, 78.7%), limb (weakness and gait disturbance, 57.4%), bulbar (dysarthria and facial palsy, 38.3%), and respiratory (dyspnea, CO_2_ retention, 40.4%) muscles (Table 1). In classical MG, the ocular symptoms are almost 100% presented. Around 70% of classical MG have limb weakness, 63% have bulbar symptoms, only 19% require ventilation at crisis, and 8% died despite being put on ventilation. The pure ocular type of irMG was observed in 14.9% of the reported cases (Table 1), which is consistent with the epidemiological data on classical MG, estimated to be around 17% (46, 47).

#### Underlying Malignancy, ICI Drug, and Onset Time During ICI Treatment

In patients with irMG, their underlying malignancies, which were treated using ICIs, included melanoma (48.9%), non-small-cell lung carcinoma (14.9%), small-cell lung carcinoma (10.6%), renal cell carcinoma (10.6%), squamous cell carcinoma (6.4%) of the bladder, thymus, and head and neck, and other malignancies such as tracheal neuroendocrine carcinoma and pulmonary pleomorphic carcinoma (8.5%; Table 1).

Most cases of irMG (89%) were treated using programmed death protein 1 inhibitors (nivolumab, 48.9%, and pembrolizumab, 40.4%). Only small proportions (11%) of cases were treated using cytotoxic-lymphocyte-associated protein 4 inhibitors (ipilimumab, 14.9%, and tremelimumab, 2.1%) or programmed death-ligand 1 inhibitors (durvalumab, 2.1%). This result was similar to a previous analysis in which higher incidences of neurological irAEs were found with monotherapy of anti-PD1 antibodies (22, 48). Most cases were treated with single ICI therapy, and only four (8.5%) received combination therapy with either ipilimumab and nivolumab or tremelimumab and durvalumab (Table 1). The onset time of irMG was primarily at approximately 1 month after the initiation of ICI therapy and usually between the second and third cycle of immunotherapy (Table 1).

#### Coexistence of irAE and the Involvement of Other Organ Systems

The coexistence of irAEs or the involvement of other organ systems was commonly observed (35/47 = 74.5%) in patients with irMG. In 35 cases of irMG with irAE, 18, 14, and three cases exhibited the involvement of one, two, and three organs in addition to neuromuscular junction. In all cases of concomitant irAEs, myositis (23/35 = 65.7%) was the most common condition, followed by myocarditis (11/35 = 31.4%), pneumonitis (5/35 = 14.3%), hepatitis (3/35 = 8.6%), and peripheral neuropathy (4/35 = 11.4%; Table 1). A total of 27 cases presented elevated creatine kinase (CK) levels, although only 23 were diagnosed with coexisting myositis. The prevalence of elevated CK levels was not different between the irMG-related mortality group and the survival/non-irMG-related mortality group (*P* = 0.768, Fisher's exact test).

#### Treatment Choice for irMG

Although the Society for Immunotherapy of Cancer has provided consensus recommendations for the management of irAEs that develop after ICI therapy, the employment of a wide variety of therapeutic strategies was observed in this review. Monotherapy and combination therapies were both reported. Maintenance immunosuppression therapy included oral prednisolone ranging from a very low dose of 3 mg QOD (21) to 100 mg QOD (15) and intravenous (IV) corticosteroid (1–2 mg/kg/day). Pulsed immunosuppression therapy included IV immunoglobulin (IVIG), plasma exchange (PE), and IV methylprednisolone therapy at doses of 500 or 1,000 mg/day (Table 1). Escalation immunotherapy, such as rituximab, was also used in the treatment of irMG. Although basal immunotherapy, such as azathioprine, was suggested for refractory cases by some authors (3), no reported cases in this review used a basal immunosuppressant to treat irMG.

### Comparisons of Patients in Different Mortality Groups: irMG-Related Mortality and Survival/Non-irMG-Related Mortality

Because of the high mortality rate of irMG, we attempted to identify the risk factors for intractable or fatal irMG. The 47 selected cases were further divided into two groups according to the cause of death: irMG-related mortality (*N* = 14) and survival/non-irMG-related mortality (*N* = 33). The major causes of irMG-related mortality were respiratory failure (86%, *N* = 12) and aspiration pneumonia due to dysphagia (*N* = 2). On the other hand, the causes of death irrelevant to irMG included bleeding of the gastrointestinal tract (*N* = 1), sepsis (*N* = 1), shock (*N* = 1), sudden cardiac arrest (*N* = 2), and recurrence of malignancy after finishing ICI treatment for a period of time (*N* = 2). The demographic data, type of malignancy, type of ICI agents, symptoms of MG, and involvement of other organs were compared, and the results in the following discussion were obtained.

#### Demographic Data, Diagnostic Tests, and Presentation of Symptoms of irMG

Data concerning demographic categories such as age and sex were similar between the irMG-related mortality group and the non-irMG-related mortality group (*P* = 0.88, *t*-test; *P* = 0.768, Fisher's exact test; respectively; Table 1). In total, nine cases had previously diagnosed MG (completely no symptom, *N* = 2; completely remitted, *N* = 2), and three resulted in irMG-related death after receiving ICI treatment. The presence of MG prior to ICI treatment increased the risk of developing irMG symptoms but was not associated with higher irMG-related mortality (Table 1).

#### Underlying Malignancy, ICI Drug, and Onset Time During ICI Treatment

The irMG-related and non-irMG-related mortality groups did not differ significantly with respect to the type of malignancy and the type of ICI applied. Of the four patients who received combination therapy, three died of irMG. In the irMG-related mortality group, the patients exhibited an earlier onset of irMG symptoms, usually within 4 weeks from the initiation of ICI therapy (*P* = 0.0302, Mann–Whitney *U*-test; one-tailed, Table 1).

#### Coexistence of irAE in Other Organ Systems

**Figure 1:**
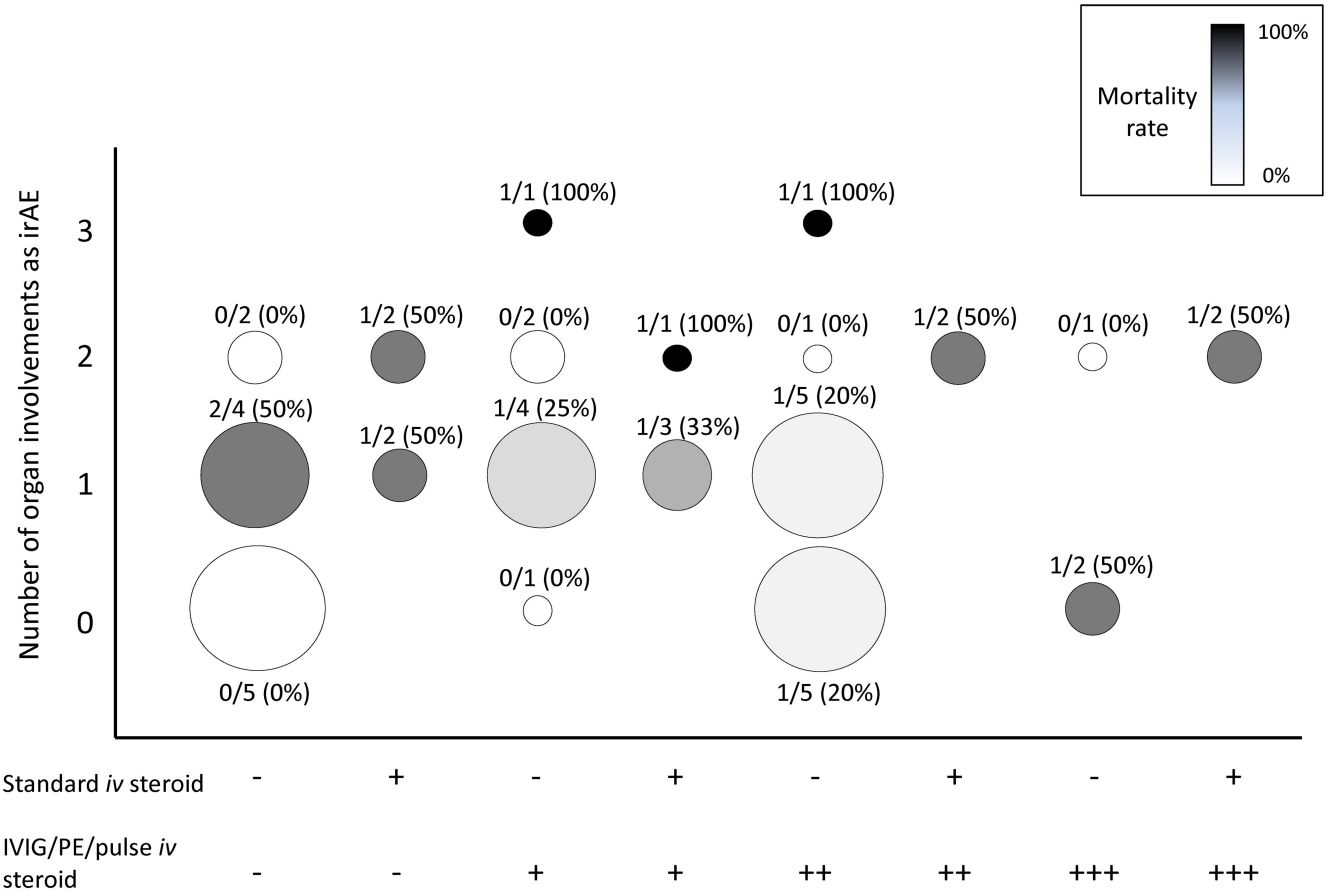
Summary of the number of organs involved as irAEs in addition to irMG, treatment strategies, and mortality rate. X axis: treatments. Number of “+,” number of treatment(s) of pulsed immunosuppression (PE, IVIG, pulse methylprednisolone) applied. Y axis: number of organs involved as irAEs. Organs for statistic included skin, gut, endocrine, lung, muscle, cardiovascular, liver, hematologic, renal, nerve, and ophthalmologic system. Circle size: total case number. Circle gray scale color, mortality rate: black, 100% mortality; white, 0% mortality. Number above each circle: numerator, case number of irMG-related mortality; denominator, total case number of each treatment strategy. PE, plasma exchange; IVIG, intravenous immunoglobulin.

The percentage of cases involving multiple organs, which suggests the extent of ICI-induced autoimmune responses, was higher in the irMG-related mortality group than in the non-irMG-related mortality group (*P* = 0.038, one-tailed *t*-test) (Table 1). In the irMG-related mortality group, two cases with three organ involvements and four cases with two organ involvements died of irMG (Figure 1). The coexisting irAE were myositis/neuropathy/nephropathy and myositis/neuropathy/myocarditis in the two with three organ involvements. The coexisting irAE were myositis/endocrine, myocarditis/gut, myocarditis/gut, and myocarditis/myositis in the four with two organ involvements. Myositis and myocarditis seem to commonly co-exist with irMG and may increase the risk of mortality. The extent of irAE may affect a physician's decision regarding the choice of therapy. Thus, a comprehensive study of the involvement of other organ systems in irMG development is necessary.

#### Choice of Treatment for irMG

The therapeutic strategies in the 47 cases varied considerably. Figure 1 summarizes the treatment strategies, number of organs involved, and the mortality rate in the 47 cases. At least eight combinations of the standard maintenance dose of corticosteroid (1–2 mg/kg/day) and pulsed immunosuppression therapies were reported. The number of cases receiving each therapy was small; thus, identifying a beneficial therapeutic strategy was difficult. The benefit of low-dose corticosteroid (1–2 mg/kg/day) was not observed in this review. In 30 cases with seropositive anti-AChR Ab results, therapies targeting the circulating autoantibodies, such as IVIG or PE, did not reduce mortality (*P* = 0.626, chi-square test). High mortality was observed in patients with involvement of multiple organs (>2) despite the use of one or two pulsed immunosuppression therapies. Multiple combination therapy was not always applied in patients with multiple irAEs, even if the mortality rate in this group of patients might have been higher. The immunosuppressant treatment for irMG can be a compromised decision regarding the patient's immunity, malignancy, and risks for opportunistic infection.

## Discussion

In summary, irMG and classical MG are clinically distinct categories of neuromuscular disorders, as evident in the differences in their demographic characteristics, pathogenesis, serology profile, response to treatment, associated complications, and prognosis. Because of the high mortality of irMG, measures to increase the vigilance of medical teams are necessary to ensure the timely identification and treatment of this condition. In addition, the diagnostic plans should be comprehensive and include the evaluation of other organ systems because the involvement of >1 organ system is a risk factor for irMG-related mortality. The differences between irMG and classical MG are summarized in the following discussion.

Classical MG has a bimodal epidemiological distribution, with female predominance in the early-onset group (age <40 years) and an equivalent incidence in both sexes in the late-onset group (age >40 years). However, patients with irMG were mostly men, and the patients were relatively older at the onset. The onset age may be related to the epidemiology of cancers treated with ICI. Compared with classical MG, the prevalence of ocular symptoms in irMG was lower, but respiratory paralysis was 2-fold higher, particularly in those who died from irMG (50%). This provides evidence which support the higher mortality rate of irMG (29.8%).

The positive rates of the RNST and the serological tests were lower in cases of irMG than in classical MG. The positive rate of RNST in classical MG has been reported to be 60% (49–51), whereas that in irMG has been reported to be 50%. The positive rate of the anti-AChR Ab test in classical MG has been reported to be 85–87% (50–52), whereas that in irMG has been reported to be 66.7%. The prevalence of anti-MuSK Ab positivity in anti-AChR Ab negative classical generalized MG varied from 37.5 to 70% (51–56), that is, anti-MuSK Ab was present in around 5–10% of overall classical generalized MG, while in our review only 5.3% of irMG patients were positive for anti-MuSK Ab. In other words, the prevalence of seronegative patients in irMG was higher than that in classical MG.

The presence of thymoma underpinning the pathogenesis is a part of classical MG but is not relevant to irMG. This result can be biased by the cancer type of patients treated with ICIs. It was possible that only a very small portion of patients with thymoma received ICI therapy compared with patients of other cancer types. Autoimmune diseases, such as systemic lupus erythematosus, rheumatoid arthritis, and thyroid disorders, have been reported to be frequently observed as comorbidities in patients with classical MG (57–60), whereas myositis, myocarditis, pneumonitis, hepatitis, and peripheral neuropathy have been more frequently observed with irMG.

The mortality rate of irMG was 29.8% in this review, which was similar to a previously reported mortality rate of 30.4% (22). By contrast, classical MG is a relatively benign disorder with low mortality (around 6%) if the patients receive timely treatment (46, 47). The difference in mortality can be attributed to multiple factors, such as the age of onset, concomitant malignancy, cancer-related complications, or response to conventional immunotherapy. IrMG is often refractory to standard therapy for classical MG. Patients with irMG-related mortality shared the following features: First, they exhibited an early onset of MG symptom, usually within the first month of ICI therapy. Second, mortality tended to be higher when multiple organs were involved. Timely diagnostic and therapeutic planning are necessary, particularly for patients with a high mortality risk. The diagnostic plans should comprehensively include the evaluation of other organ systems in addition to the performance of traditional diagnostic studies such as RNST, pyridostigmine/edrophonium test, and serology tests for anti-AChR Ab and anti-MuSK Ab. Regarding the seronegative irMG, studies for less common autoantibodies, such as anti-voltage-gated potassium channel Ab, or anti-striatal antibody, including anti-titin Ab, anti-actin Ab, and anti-myosin Ab, may provide more information (40, 43).

Respiratory paralysis is the major cause of death in irMG. In 33 surviving/irMG-irrelevant mortality patients, although 12 experienced MG-related respiratory symptoms, six recovered from immune therapy and non-invasive positive pressure ventilation support. Only three needed an artificial airway. However, in 14 patients with irMG-related mortality, seven experienced MG-related respiratory symptoms, and invasive ventilation was recommended for six patients. Among the six patients who needed an artificial airway, four did not complete the entire treatment course of MG crisis and finally received hospice care. It seemed that the severity of respiratory paralysis, which varied from the demand of non-invasive to invasive ventilation support, might largely determine the outcome. The benefit of early invasive ventilation support was not seen here, which can be due to the limited number of cases.

The presence of MG prior to ICI treatment increased the risk of developing irMG symptoms but was not a risk factor for irMG-related mortality (Table 1). Although ambiguity exists while defining the re-appearance of MG symptom as an acute exacerbation of classic MG or a newly developed irMG, particular attention paid to MG patients who were exposed to ICI was required based on the following reasons: Firstly, the presence of MG prior to ICI treatment can be a risk of developing irMG. Secondly, the clinical presentations, such as the extent of muscle involved, disease progression, and serology, can be quite distinct from the remote, completely remitted MG (35). Thirdly, the mortality rate of this group of patients was 30%, which was much higher than that of classical MG. Since 1969, after immunosuppressant, IVIG, and plasma exchange were introduced as standard treatments for MG, the mortality rate of classical MG was decreased to around 6% (46). Fourthly, the cancers treated with ICIs in these nine cases were melanoma (*N* = 7), tracheal neuroendocrine cancer (*N* = 1), and renal cell carcinoma (*N* = 1). None was reported to have thymic lesion, which was distinct to classical MG. The MG symptoms that developed following ICI treatment were not as “benign” as those of classical MG and undoubtedly needed to be recognized promptly and treated more aggressively (35). For patients with pre-existing MG that require ICI treatment for end-stage malignancy, meticulous evaluation should be made to weigh the benefit of cancer treatment and the risk of pre-existing MG recurrence or developing irMG as both conditions bring mortality to the patient (61, 62).

Occasionally, irMG co-existed with ir-myositis. Both present with limb weakness and even respiratory failure due to diaphragmatic involvement (63, 64). Carefully distinguishing the two similar conditions was required because the co-existence of the two irAEs may increase the mortality rate (the mortality rate in irMG with ir-myositis is 35%; in irMG without myositis, it is 25%). Comprehensive measures including serum creatine kinase levels, muscle ultrasound, diaphragm nerve conduction study, electromyography, single muscle electromyography, and even muscle biopsy may help to confirm the diagnosis (41). Moreover, ir-myocarditis also needed to be studied in patients with irMG. Both may lead to ventilation dependence or a fatal event (65–69). Early detection and treatment for vital organ dysfunction such as timely intubation for respiratory failure, pacemaker implantation for fatal arrhythmia, and vasopressor and even extracorporeal membrane oxygenation for cardiogenic shock should be considered if clinically needed and available in addition to immunosuppressants (70).

Although the Society for Immunotherapy of Cancer has provided recommendations, no published study designed to investigate the treatment of irAE is currently available. In this review, the therapeutic strategies of the 47 cases varied widely. For irMG initial treatment, no unanimous conclusion could be drawn from the big variety of published reports. Both those who are for and those who are against using steroid monotherapy for neurological irAE are present (71–74). There were plans to start with a high dose methylprednisolone or IVIG (72), to start plasma exchange and IVIG initially regardless of clinical severity (73), and, alternatively, to use immunosuppressants like mycophenolate, methotrexate, cyclophosphamide, rituximab, natalizumab, bortezomib, and even tacrolimus for refractory cases (74), but whatever immunosuppressant is used for treating neurological irAE, the clinician should always be aware of opportunistic infection (75). After recovering from irAE, re-challenging with another class of ICI may be considered because neurological irAE caused by one class of ICIs is not necessarily provoked by another class of ICIs (75).

The information obtained from the collected case reports represents only a small fraction of the actual number of cases worldwide. The sampling bias is a limitation in this review because we could not estimate the number of unreported cases. The small sample size in this review also limited the statistical power and reduced the potential to extrapolate from the results. Although irMG is rare, we contend that the number of patients with irMG is poised to rapidly increase with the increasing use of ICIs. The current study, despite its limitation in sample size, provides an overview of this life-threatening condition and contributes to the field by increasing the vigilance of medical teams to ensure timely diagnosis and treatment of this condition.

Although the therapeutic effects of ICIs have drawn considerable attention recently, potentially devastating irAEs, such as irMG, should not be overlooked. Early identification always benefits patients undergoing ICI treatment who develop new neurological signs, particularly in the early phase of ICI therapy. A complete study focusing on the involvement of other organ systems is required because the involvement of organ systems might be related to mortality. Early identification of patients at a high risk of mortality may result in rapid and timely interventions and promote the early preparation of a comprehensive treatment plan. With the increasing use of ICIs, additional therapeutic studies concerning irMG in the future are needed to minimize the irAE-related mortality and increase the safety of patients with cancer who are undergoing immune therapy.

## Author Contributions

Y-TH designed the study, analyzed the data, and drafted the manuscript. W-CL analyzed the data. Y-PC and W-CS revised the draft. Y-TS approved the final version of the manuscript on behalf of all the authors.

## Conflict of Interest

The authors declare that the research was conducted in the absence of any commercial or financial relationships that could be construed as a potential conflict of interest.
