# Supplementary material for: Immune Checkpoint Inhibitor-Induced Myasthenia Gravis
Source: Front Neurol. 2020 Jul 16;11:634. doi: 10.3389/fneur.2020.00634 (PMC7378376; doi:10.3389/fneur.2020.00634)
Supplement: Supplementary file 1 [file Table_1.DOCX]

| Suppl Table1: Reported cases of irMG | | | |  | |  | | | | |  | | | | |  | | | | | |  | | | | | | | | | | | |  |  |
| --- | --- | --- | --- | --- | --- | --- | --- | --- | --- | --- | --- | --- | --- | --- | --- | --- | --- | --- | --- | --- | --- | --- | --- | --- | --- | --- | --- | --- | --- | --- | --- | --- | --- | --- | --- |
|  | | | **Symptom** | **Onset time** | | **Diagnostic exams (Positive = +, Negative = -, not check = blank)** | | | | | **Drug choice**  **(Yes = +, No = blank)** | | | | | **Treatment (Yes = +, No = blank, unsure = ?)** | | | | | | **Associated organ involvement**  **(Yes = +, No = blank)** | | | | | | | | | | | | **CK** | **Outcome** |
| **Reference** | **Age & Sex** | **Malignancy** | **Ocular = O**  **Bulbar = B**  **Generalized = G**  **Respiratory = R** | **Week** | **Cycle** | **ice packing** | **Neostigmine test** | **AChR** | **MuSK** | **RNST** | **Ipilimumab** | **Tremelimumab** | **Nivolumab** | **Pembrolizumab** | **Durvalumab** | **IVIG** | **PE** | **Methyprednisolon** | **Prednisone** | **Pyridostigmine** | **Rituximab** | **Skin** | **Digestive organ** | **Endocrine** | **Respiratory** | **Skeleton** | **Heart** | **Blood** | **Muscle** | **Nerve** | **Kidney** | **Eye** | **Organ_sum** | **CK level_value** | **Mortality = 1**  **Alive = 0, unsure=?** |
| Zimmer et al. | 69F | Melanoma | **O, G** | 10 | 2 |  |  | **-** |  |  |  |  |  | + |  |  | + | + |  | + |  |  |  |  |  | + |  |  |  |  |  |  | 1 | >300 | 1 |
| Makarious et al. | 85F | Melanoma | **O** | 4.5 | 2 |  |  | **-** | **-** |  |  |  |  | + |  | + |  |  | + | + |  |  |  |  |  |  | + |  |  |  |  |  | 1 | NA | 1 |
| March et al. | 63M | Melanoma | **O, B, R** | 2 | 1 |  |  | **-** | **-** |  |  |  |  | + |  | + | + | + | + | + |  |  |  |  |  |  |  |  |  |  |  |  | 0 | >300 | 1 |
| Gonzalez et al. | 71F | Uterine carcinosarcoma | **O, B** | 12 | 4 |  |  | **-** | **-** |  |  |  |  | + |  |  |  |  | + | + |  |  |  |  |  |  |  |  | + |  |  |  | 1 | 1200 | ? |
| Nguyen et al. | 81M | Melanoma | **O** | 11 | 3 | + |  | **-** |  |  |  |  |  | + |  |  |  |  | + |  |  |  |  |  |  |  |  |  |  |  |  |  | 0 | NA | 0 |
|  | 86F | Melanoma | **O, B** | 7 | 2 |  |  | **-** | **-** | **-** |  |  |  | + |  |  |  | + | + |  |  |  |  |  |  |  |  |  |  |  |  |  | 0 | NA | 0 |
| Alnahhas et al. | 84F | Melanoma | **O, B** | 4 | 2 |  |  | + | **-** |  |  |  |  | + |  | + |  |  | + | + |  |  |  |  | + |  | + |  |  |  |  |  | 2 | NA | ? |
| Polat et al. | 65M | NSCLC | **O** | 8 | 3 |  |  | **-** | **-** | **-** |  |  | + |  |  |  |  |  |  | + |  |  |  |  |  |  |  |  |  |  |  |  | 0 | NA | 0 |
| Sciacca et al. | 81M | NSCLC | **O, G** | 6 | 3 |  |  | + |  | **-** |  |  | + |  |  |  |  |  | + |  |  |  |  |  |  |  |  |  |  |  |  |  | 0 | NA | 0 |
| Chang et al. | 75M | SCC | **O, B, G, R** | 6 | 2 |  |  | + | **-** | + |  |  | + |  |  | + |  |  |  | + |  |  |  |  | + |  |  |  | + |  |  |  | 2 | 1587 | ? |
| Lopez et al. | 65M | RCC | **O, G, R** | 3 | 2 |  |  | + |  |  |  |  | + |  |  | + |  |  |  |  |  |  |  |  |  |  |  |  | + | + | + |  | 3 | 6321 | 1 |
| Shirai et al. | 81F | Melanoma | **O, G, R** | 2 | 1 |  | + | + |  | **-** |  |  | + |  |  |  |  | + |  |  |  |  |  | + |  |  |  |  | + |  |  |  | 2 | 8729 | 1 |
| Kimura et al. | 80M | Melanoma | **R** | 2 | 1 |  |  | + | **-** |  |  |  | + |  |  | + | + | + | + |  |  |  |  |  |  |  | + |  | + |  |  |  | 2 | 7740 | 0 |
| Liao et al. | 70F | Melanoma | **O, B, G** | 4 | 2 |  |  | + |  | + | + |  |  |  |  |  | + | + |  |  |  |  |  |  |  |  |  |  | + |  |  |  | 1 | 1200 | 0 |
| Johnson et al. | 69F | Melanoma | **O, B, G, R** | 6 | 3 |  |  | + |  | + | + |  |  |  |  |  | + | + |  | + |  | + |  |  |  |  |  |  |  |  |  |  | 1 | NA | 0 |
|  | 73F | Melanoma | **G, R** | 3 | 2 |  |  | + |  |  | + |  |  |  |  |  |  |  | + | + |  |  |  |  |  |  |  |  |  |  |  |  | 0 | NA | ? |
| Montes et al. | 74M | Melanoma | **O, G, R** | 6 | 3 |  | + | **-** | **-** | + | + |  |  |  |  |  |  |  | + | + |  | + |  |  |  |  |  |  |  |  |  |  | 1 | NA | 0 |
| Antonia et al. |  | NSCLC |  | 2 | 1 |  |  |  |  |  |  | + |  |  | + | **?** | **?** | **?** | **?** | **?** | **?** |  |  |  |  |  |  |  |  |  |  |  | 0 |  | 1 |
| Loochtan et al. | 70M | SCLC | **O, R** | 2 | 2 |  |  | + |  | + | + |  | + |  |  | + | + | + | + |  |  |  | + |  |  |  | + |  |  |  |  |  | 2 | NA | 1 |
| Lau et al. | 75M | Melanoma | **O, B, G** | 5 | 2 |  |  | + |  |  |  |  |  | + |  | + |  | + |  |  |  |  |  |  |  |  |  |  |  |  |  |  | 0 | NA | 0 |
| Zhu et al. | 59 | Melanoma | **B** | 9 | 3 |  |  | **-** | **-** | + |  |  |  | + |  | + | + |  | + |  |  |  |  |  |  |  |  |  |  |  |  |  | 0 | NA | 0 |
| Phadke et al. | 75M | Melanoma | **B, R** | 6 | 2 |  |  | + | **-** |  |  |  |  | + |  | + | + |  | + | + | + |  |  |  |  |  |  |  |  |  |  |  | 0 | NA | ? |
| Maeda et al. | 79M | Melanoma | **O, B** | 9 | 3 |  |  | + |  |  |  |  | + |  |  |  |  |  | + |  |  |  |  |  |  |  |  |  | + |  |  |  | 1 | 1627 | 1 |
| Tan R.Y.C et al. | 45M | NSCLC | **O, R** | 2 | 1 |  |  | + |  | **-** |  |  | + |  |  | + |  | + |  | + |  |  |  |  |  |  |  |  | + |  |  |  | 1 | >300 | 0 |
| Fukasawa et al. | 69F | NSCLC | **O, G** | 7 | 3 |  |  | + |  |  |  |  | + |  |  |  |  | + |  |  |  |  |  |  |  |  | + |  |  |  |  |  | 1 | 1156 | 0 |
| Alnahhas et al. | 84M | Melanoma | **O, B, G** | 4 | 2 |  |  | + | **-** |  |  |  |  | + |  | + |  | 0 | + | + |  |  |  |  | + |  | + |  |  |  |  |  | 2 | NA | ? |
| Chen et al. | 65M | SCLC | **O, G** | 7 | 3 |  |  | **-** |  | **-** |  |  | + |  |  |  |  | + |  | + |  |  |  |  |  |  |  |  | + |  |  |  | 1 | 2216 | 1 |
|  | 57M | SCLC | **O, G, R** | 6 | 2 |  |  | + |  | **-** | + |  | + |  |  |  |  | + | + | + |  |  |  |  |  |  |  |  | + | + |  |  | 2 | 2682 | 0 |
| Rota et al. | 72F | RCC | **G** | 2 | 1 |  |  |  |  | + |  |  | + |  |  | + |  | + |  |  |  |  |  |  |  |  |  |  |  |  |  |  | 0 | NA | 0 |
|  | 71M | RCC | **G** | 2 | 1 |  |  |  |  | + |  |  | + |  |  | + |  | + |  |  |  |  |  |  |  |  | + |  | + | + |  |  | 3 | NA | 1 |
| Mitsune et al. | 62F | tracheal neuroendocrine carcinoma | **O, B** | 4 | 2 |  |  | **-** | + |  |  |  | + |  |  |  |  | + |  |  |  |  |  |  |  |  |  |  | + |  |  |  | 1 | 14229 | 0 |
| Mehta et al. | 73M | RCC | **G** | 2 | 2 |  |  | + |  |  |  |  | + |  |  | + | + |  |  | + |  |  |  |  |  |  |  |  | + |  |  |  | 1 | 8950 | 0 |
| Earl et al. | 74M | Melanoma | **O, B, G** | 4 | 2 |  |  | + | **-** | + |  |  |  | + |  | + | + |  | + | + |  |  |  |  |  |  |  |  |  |  |  |  | 0 | NA | 1 |
| Kang et al. | 75M | SCC | **O, B, G** | 3 | 1 |  |  | + | **-** |  |  |  | + |  |  |  | + | + | + | + |  |  |  |  |  |  |  |  | + |  |  |  | 1 | 2593 | 1 |
| Onda et al. | 73M | NSCLC | **O** | 3 | 1 |  | - | **-** | **-** | **-** |  |  |  | + |  |  |  | + | + |  |  |  |  |  |  |  |  |  | + |  |  |  | 1 | 7311 | 0 |
| Tozuka et al. | 82M | pulmonary pleomorphic carcinoma | **O** | 12 | 3 |  |  | + |  |  |  |  |  | + |  |  |  |  |  | + |  |  |  |  | + |  |  | + |  |  |  |  | 2 | NA | 0 |
| Algaeed et al. | 73M | Melanoma | **O** | 3 | 1 |  |  | + |  |  |  |  |  | + |  | + | + |  | + |  |  |  |  |  |  |  |  |  |  |  |  |  | 0 | NA | 0 |
| Crusz et al. | 78M | Melanoma | **O, B, G** | 4 | 2 |  |  | + |  |  |  |  |  | + |  | + | + | + |  |  | + |  |  |  |  |  | + | + |  |  |  |  | 2 | 1109 | 0 |
| Hibino et al. | 83M | SCLC | **O, G** | 4 | 2 | + | + | **-** | **-** | **-** |  |  |  | + |  |  |  |  | + | + |  |  | + |  |  |  |  |  | + |  |  |  | 2 | 4361 | 0 |
| Huh et al. | 34F | SCC | **O, G** | 5 | 4 |  |  | + |  | **-** |  |  |  | + |  | + |  | + | + |  |  |  |  |  |  |  |  |  | + |  |  |  | 1 | 2126 | 0 |
| Möhn N et al. | 87F | Melanoma | **O, G** | 4 | 1 |  |  | + |  |  |  |  | + |  |  |  |  |  | + |  |  |  |  |  |  | + | + |  |  |  |  |  | 2 | >4000 | 1 |
|  | 82M | Melanoma | **O, G** | 6 | 1 |  | - | + |  |  |  |  | + |  |  | + |  | + |  |  |  |  |  |  |  |  |  |  | + | + |  |  | 2 | >2000 | 1 |
| Shotaro Nakanishi et al. | 78M | RCC | **R** | 4 | 2 |  |  | + |  |  |  |  | + |  |  |  |  |  |  |  |  |  |  |  |  |  |  |  | + |  |  |  | 1 | >6000 | 1 |
| Noda et al. | 77F | NSCLC | **O, B, G, R** | 7 | 1 |  |  | + |  | **+** |  |  |  | + |  |  | + | + | + |  |  |  |  |  |  | + |  |  | + |  |  |  | 2 | >6000 | 0 |
| Kenji et al. | 78M | Bladder cancer | **G, P** | 2 | 1 |  |  | + |  | **-** |  |  |  | + |  |  |  | + | + |  |  |  |  |  |  |  |  |  | + |  |  |  | 1 | >2000 | 0 |
| Becquart et al. | 75F | Melanoma | **O** | 5 | 3 | + |  | - |  | **-** |  |  | + |  |  |  |  |  |  |  |  |  |  |  |  |  |  |  |  |  |  |  | 0 |  | 0 |
| Mahdieh et al. | 78F | Melanoma | **O, B, G, R** | 1 | 1 |  |  |  |  |  | **+** |  | + |  |  | **+** | + | + |  | + |  |  |  |  |  |  | + |  | + |  |  |  | 2 | >9000 | 1 |
| Yoshikazu et al. | 76F | NSCLC | **O, B, G, R** | 3 | 2 |  |  | + | - |  |  |  | + |  |  | + | + |  | + |  |  |  |  |  |  |  |  |  | + |  |  |  | 1 | 6566 | 0 |
|  | 78F | SCLC | **O, B, G, R** | 2 | 1 |  | + | **-** | **-** | + |  | + |  |  | + |  | + | + |  | + |  |  | + |  |  |  | + |  |  |  |  |  | 2 | NA | 1 |
